# Odontalgia*Read before the Dallas Medical and Surgical Society, February 2, 1907.

**Published:** 1907-02

**Authors:** Price Cheaney

**Affiliations:** Dallas, Texas


### TEXAS MEDICAL JOURNAL.

Established July, 1885.

F. E. DANIEL, M. D., - - - - Editor, Publisher and Proprietor.

PUBLISHED MONTHLY.—SUBSCRIPTION $1.00 A YEAR.

VOL. XXII. AUSTIN, FEBRUARY, 1907. No. 8.

The publisher is not responsible for the views of contributors.

For Texas Medical Journal.

### Odontalgia.*

BY PRICE CHEANEY, B. S., M. D., D. D. S., DALLAS, TEXAS.

Toothache is generally regarded as a little thing, but—did you ever have it? If you have, you will realize that it is one of the biggest little things which this dear old world contains. At any rate, that is what the patient thought who called you, after you supposed you had finished your day's work and retired to get a good night's rest, and without giving you any particulars told you to come at once. You doubtless thought, from the urgency of the call, that it was a case in which you would have to hustle if you got there before the stork did, and was disgusted when you found that it was only a case of toothache, but what are you going to do about it?

I know from experience, as suggested in a former paper before this body, that the medical schools evidently don't think it a matter of enough importance to give anything like exhaustive information regarding the diseases of the oral cavity, and, because this is a practical thing, one which you may be called upon to look after any day, I have presumed to take up a few minutes of your valuable time in discussing this little thing. But after all, life is made up of little things, and every practitioner of any experience knows that sometimes the retention of the practice of his best families has hinged on being able to attend to the little things.

Histologically speaking, a tooth is composed of four parts. First, an outer covering of enamel, the hardest of all animal substances, which has the same relation to the tooth that the case-hardened

*Read before the Dallas Medical and Surgical Society, February 2, 1907.

steel covering of a burglar-proof safe has to the interior of the safe —a protective. It is composed of dense hexagonal rods — the enamel rods—giving a line of cleavage from the circumference to the center, covered by a hard, smooth surface—Nasmyth's membrane—the remains of the enamel organ, which is literally "the skin of the teeth." So far as can be demonstrated, the enamel is devoid of nervous structure of any kind, is incapable of conducting sensation of any kind, and is purely an organ of protection for the softer structures underneath.

The next structure, the dentine, is less dense and contains thousands of tubuli radiating from the pulp chamber to the periphery, and containing odontoblastic matter which possesses the property of conveying calcareous materials from the inside outwards, and capable of conveying sensation from the pulp. Histologists, I believe, do not class this as true nerve tissue, although some describe it as prolongations of nerve terminals. Whether this odontoblastic matter is in any way true nervous material or not, matters not as far as the purpose of this paper is concerned. Suffice it to say that it has the property of conducting sensation and carrying lime salts for the hardening of the tooth structure. It is, however, very hard to convince a patient that anything which is capable of such exquisite sensitiveness in the preparation of a cavity has not, to say the least, some of the characteristics of a nerve.

Next in order we come to *the pulp* itself, situated in the central cavity of the tooth, which is called the pulp chamber. This pulp, or the nerve, as it is called by the laity, is composed of a nerve, an artery and a vein, all anastomosed together into a network which renders it, macroscopically, one structure, but when seen under a high-power microscope, forms a beautiful picture, showing each of the component parts as separate.

The pulp in childhood occupies a much larger space proportionately than in old age—continually contracting as it builds up layer after layer of osteodentine around itself, so that in old age it is sometimes almost entirely obliterated.

The fourth and last of the structures of which a tooth is composed is *the cementum* or crusta petrosa, or outer layer of the root structure. This is true bone substance, having its regular system of lacunae and canalicula similar to other bones, and receives its life and nourishment from a covering of periosteum—the *pericementum* —while the dentine receives its life and nourishment from the pulp. This explains why a so-called dead tooth does not become an irritant and become exfoliated like necrosed bone, for the pulp may be dead and the cementum remain healthy through the blood supply from the periosteal membrane, so that the term "dead tooth," as applied to the entire tooth, is a misnomer.

Having thus briefly considered the anatomical structures involved, let us consider the pathological conditions which lead up to odontalgia, or toothache proper.

The most common etiological factor leading up to toothache is caries or decay of tooth structure, and this grows, primarily, out of uncleanliness.

Of no other part of the human system can it be said more truthfully "that cleanliness is next to Godliness" than of the mouth and teeth. It is not overstating it to say that if teeth could be kept absolutely clean they would never decay, but with the soft, sticky foods which are a result of our modern civilization, it is next to impossible to maintain a condition of absolute cleanliness.

The interdental spaces between the teeth and the interstices in the sulci on the masticating surfaces afford an inviting place for the accumulation of particles of food which decomposes under the conditions of heat and moisture which the mouth provides, and become breeding places for colonies of micro-organisms whose ptomains act chemically upon the surfaces of the teeth, dissolving the outer surface so that a cavity is soon formed which enlarges more rapidly after the dense enamel surface is penetrated until the tooth becomes at first sensitive to thermal changes, as only a thin layer of dentine is left to protect the delicate pulp. Sweets and sours give an occasional pang, and finally we have, as decay progresses, a total exposure of the pulp. We now have *pulpitis,* or inflammation of the pulp, from contact of external irritants and thermal changes.

Every breath of air, every drink of cold water, gives excruciating pain, and, as in other parts of the body where there is irritation of any kind, nature sends an increased blood supply which causes a swelling of the delicate structure of the pulp against the hard, unyielding walls of the pulp chamber, causing pressure upon the nerve filaments, producing a more permanent character to the pain. Finally, the congestion becomes so intense that stasis of the blood current takes place, followed by gangrene of the pulp. This is followed by a period of quiet and relief from pain—sometimes for months—until decomposition of the pulp sets up, and mephitic gases produced thereby, escape at the apical foramen and cause an irritation which grows into inflammation of the periosteal membrane surrounding the root, and we have a case of *pericementitis* or a periostitis similar in character to that found in a bone felon.

Again we have the same succession of changes which we found in our case of pulpitis: irritation, inflammation, congestion, stasis, death of tissue and finally pus formation, when the last stage, or *alveolar abscess,* appears.

Accompanying these changes we have symptoms which are characteristic in each stage. I have already mentioned the sensitiveness to thermal changes in pulpitis. The pain of pulpitis is a sharp, cutting pain, but when the pulp dies and the expansion of gases of decomposition causes an irritation of the periosteum there is an increased blood supply to the periosteum which causes a swelling of the membrane and consequent thickening, and, as the tooth is encased in a bony socket, this forces the tooth outward and causes a feeling of elongation and sensitiveness to pressure which did not exist during the pulpitis. The patient will complain of a dull, dead, throbbing pain, every beat of the heart causing added pressure to the sensitive membrane and contact with the opposing teeth being very painful. If, during this stage, you palpate the tooth between your fingers it will seem very loose, while with pulpitis it is generally as firm as the rest of the teeth and closing the teeth does not cause pain.

When the stage of pericementitis is passed and it becomes a case of abscess, the symptoms vary so little that it is frequently hard to diagnose the difference, but, when the surrounding tissues become involved and swelling of the face takes place, or there is a rise in temperature, then the picture is easily recognized.

This, in brief, is the pathology of toothache as the term is generally understood, and while we have this picture clearly before us I will say a few words about its treatment.

When the patient complains of a quick, jumping pain with extreme sensitiveness to heat or cold or sweets, the tooth firm and no additional pain upon pressure from other teeth, then probably we have a case of pulpitis, and relief can be given by applying a pledget of cotton to the cavity of decay saturated with oil of cloves, laudanum, creosote, carbolic acid or chloroform. However, it is well to steer clear of carbolic acid and creosote, as great damage is often done to the oral mucous membranes by the escharotic effect of these remedies, even when skillfully applied. It is well to bear in mind, also, to be careful not to use a large pledget of cotton, as the pressure upon the pulp may cause great pain.

When the patient complains of a dull, dead pain with tenderness upon contact with the occluding tooth caused by the tooth being longer than the others (a characteristic expression), then you probably have a case of pericementitis, caused by gases of decomposition from a dead pulp, and the first and logical thing to do is to open into the pulp chamber with a small drill and provide for a relief of the tension thereby. This, together with the use of counter-irritants applied to the gums, will give relief in a short time.

The counter-irritant in most common use by the dental profession for this purpose is a combination of equal parts of tincture of aconite root, tincture of iodine and chloroform. However, a remedy which is nearly always at hand and which serves an excellent purpose, is spirits of camphor. These remedies are applied by means of a mop made by twisting a little cotton on a wooden toothpick and painting the gum around the affected tooth.

When the symptoms of pericementitis have a history extending over any length of time, with increasing intensity, then the possibility of a blind abscess suggests itself, and when the surrounding tissues become involved and swelling of the face with temperature accompanies it, then we are certain that we have to contend with an alveolar abscess, and the evacuation of pus is necessary for relief.

The treatment of an abscess, as far as the immediate relief of the patient is concerned, is a matter of discharge of pus and drainage. This is a simple matter when the pus has made an opening for itself through the alveolar process and fluctuation of pus can be felt by palpation, but with a blind abscess, where the pus is still confined within the socket, it is different and sometimes a difficult problem. Sometimes, especially in upper teeth, simply opening into the pulp cavity will allow drainage through the roots of the tooth, but when this fails, then it becomes necessary to drill through the alveolar process at the point nearest the apical part of the root and to follow up with such treatment as would be indicated with an abscess in any other part of the body.

So far, I have confined myself to the ordinary and typical cases of toothache. It might be well, however, before closing this paper, to refer to reflex pain in teeth caused by irritation in some other point. Frequently the pain will seem to be in one tooth when, in reality, the point of irritation is in another tooth, possibly in the opposite jaw. It is a frequent occurrence for a patient to come in, say, with a pain in a lower biscupid tooth which examination proves to be all right. In such cases, I often find the trouble in an upper wisdom tooth. The only rule for such cases is, that if the tooth seemingly affected shows no visible cause for the trouble, just look at the other teeth on the same side of the mouth (for reflected pain never crosses the median line), and you will be pretty apt to find another tooth which is responsible for the trouble.

Frequently teeth affected by pyorrhea alveolaris will have all of the symptoms of pericementitis or alveolar abscess, and might in some cases mislead, but this is generally easily recognized by the peculiar purplish color of the gums and the extreme looseness of the tooth, together with the fact that pressure on the gums causes pus to exude at the gingival margins. The fact that pyorrhea affects perfectly sound teeth in the majority of instances, will aid in your diagnosis of this trouble. Being an affection of the alveolar socket, temporary relief can be obtained by carrying some soothing preparation into the pyorrhea pockets with a small abscess syringe (and, for this purpose, there is nothing better than camphophenique) and counter-irritation applied to the gums as outlined in the treatment of pericementitis, accompanied by a lithia or salycilate of soda general treatment, for the uric acid diathesis which underlies it, but, as Rudyard Kipling says, "That's another story."

Another and perhaps the most difficult cause to recognize of toothache is what is known as pulp nodules. This consists of the formation of nodules of osteo-dentine in the body of the pulp, or sometimes of ossification of the entire pulp. I have already referred to the function of the pulp in carrying lime-salts for the hardening of the dentine, and of the fact that the pulp is constantly growing smaller by building around itself layers of osteo-dentine. Now, in the process of being carried to the dentine, sometimes there is an interference with the process by which a portion of the lime-salts are stopped in the body of the pulp and forms a nucleus for a formation which constantly increases. In this case, the presence of the nodule acts as an irritant and causes neuralgia, which are at times very difficult to locate, because it frequently occurs in teeth which are not even decayed, and to all appearances are perfect. Sometimes it seems that nature is making an effort to throw out a wall of bone to protect itself against the ravages of decay which has just caused enough irritation to produce an increased activity in the deposition of lime-salts. Sometimes this process is comparatively painless and the first attention which it attracts to itself is by a tooth exhibiting symptoms of pulpitis and pericementitis at the same time, and upon opening into a tooth of this kind it is found to contain both a living and a dead pulp—this in teeth with more than one root. The pathological history, in such cases, is this: A nodule of osteo-dentine is formed in one root of the tooth, which finally destroys the vitality in the root by cutting off its circulation while the remainder of the tooth receives its blood supply from the other roots and retains its vitality. No trouble occurs, unless it is of a remote neuralgic character, which is not traced to its proper source until gases of decomposition form in the dead root, and, by their expansion, cause not only a severe pulpitis in the remaining portion of the pulp from pressure, but also cause inflammation of the pericementum by being forced through the apical foramen, so that we have a tooth sensitive to heat and cold, and, at the same time, elongated and sore to pressure. Of course, in such a case, relief can only be obtained by opening into the pulp chamber and devitalizing the remainder of the pulp.

In a former paper I referred to the possibility of neuralgias and reflected pains from contiguous and even remote parts and organs, but, for the purpose of this paper, the discussion of odontalgia or toothache proper, the foregoing is, I believe, all that is necessary to cover the case.

